# Response Evaluation of Choroidal Melanoma After Brachytherapy Using Diffusion-Weighted Magnetic Resonance Imaging (DW-MRI): Preliminary Findings

**DOI:** 10.3389/fonc.2020.00825

**Published:** 2020-05-19

**Authors:** Flávia B. C. S. N. Bitencourt, Almir G. V. Bitencourt, Martha M. M. Chojniak, Juliana O. Souza, Douglas G. Castro, Antônio Cassio A. Pellizzon, Rubens Chojniak

**Affiliations:** ^1^Diagnostic Center in Ophthalmology, Fleury Medicina e Saúde, São Paulo, Brazil; ^2^Imaging Department, A.C.Camargo Cancer Center, São Paulo, Brazil; ^3^Ophthalmology Department, A.C.Camargo Cancer Center, São Paulo, Brazil; ^4^Radiation Oncology Department, A.C.Camargo Cancer Center, São Paulo, Brazil

**Keywords:** eye neoplasms, melanoma, diffusion magnetic resonance imaging, brachytherapy, response evaluation criteria in solid tumors

## Abstract

**Purpose:** To evaluate the role of diffusion-weighted magnetic resonance imaging (DW-MRI) in the assessment of therapeutic response in patients with choroidal melanoma treated with brachytherapy.

**Materials and Methods:** We performed a prospective, unicentric study which included patients with choroidal melanoma and indication for brachytherapy. Three DW-MRI examinations were proposed for each patient, one before and two after treatment. The apparent diffusion coefficient (ADC) value was calculated on DW-MRI and compared with local tumor control assessed by ophthalmologic follow-up.

**Results:** From 07/2018 to 06/2019, 19 patients were recruited, 13 of whom underwent follow-up examinations. Patients' ages ranged from 24 to 78 years and 52.9% were male. At the ocular ultrasound, the mean tumor thickness and diameter were 6.3 and 11.5 mm, respectively. Two patients (15.4%) showed signs of tumor progression during follow-up (7 and 9 months after treatment). There was no statistically significant difference in tumor size between MR before and after treatment, however, there was a significant reduction in mean ADC in patients with progression (*p* = 0.02).

**Conclusion:** DW-MRI is a promising method for monitoring patients with choroidal melanoma; reduction in the mean ADC values between pre-treatment MRI and the first post-treatment MRI may be related to the lack of response to brachytherapy and increased risk of disease progression.

## Introduction

Despite being the most common primary intraocular tumor in adults, choroidal melanoma is a rare disease, with high morbidity and mortality (1, 2). Patients who do not undergo local treatment have higher mortality in 5 years when compared to treated patients (3). Brachytherapy has been used in the treatment of choroidal melanoma because it allows for conservation of the eyeball and provides a similar prognosis to patients treated by enucleation (4–9). However, up to 50% of patients may progress with distant metastasis regardless of the local treatment (10). Failure in local tumor control after brachytherapy is relatively rare (11, 12), but patients who experience tumor recurrence or progression have a higher risk of developing distant metastases (13). For this reason, it is essential to monitor these patients closely after treatment.

Currently, the most common imaging method for assessing choroidal melanoma is ocular ultrasound. This method assists in confirming the diagnosis, and in estimating the size and location of the tumor for treatment planning and follow-up. However, when assessing therapeutic response after brachytherapy, the reduction in tumor size by ultrasonography is not apparent until six months after treatment (14, 15). Magnetic resonance imaging (MRI) is another available imaging method for assessing tumor size, presence of extra-scleral extension, or associated retinal and choroidal detachments, which could also be used for brachytherapy planning and control (16–18). However, this method is not routinely used due to its high cost and time of examination, in addition to limited availability in some centers.

The diffusion-weighted MRI (DW-MRI) is a functional MR sequence that assesses the mobility of water molecules, which is closely related to tissue cellularity and integrity of cell membranes. DW-MRI has been used as an important parameter for diagnosis, prognosis and, mainly, for assessing response to treatment in several types of cancer, including head and neck tumors (19–21). Some authors have demonstrated that DW-MRI can be useful in the evaluation of intraocular tumors, including in cases of choroidal melanoma (17, 22–24). Recent studies have suggested that DW-MRI can be used to assess early therapeutic response in patients undergoing proton beam radiotherapy (25–27). Thus, we believe that DW-MRI can also be applied to patients undergoing brachytherapy, allowing early identification of those who have failed local treatment, making it possible to reduce mortality in these cases through early diagnosis and retreatment.

The aim of this study was to evaluate the role of DW-MRI in the assessment of therapeutic response in patients with choroidal melanoma treated with brachytherapy.

## Materials and Methods

**Figure 1:**
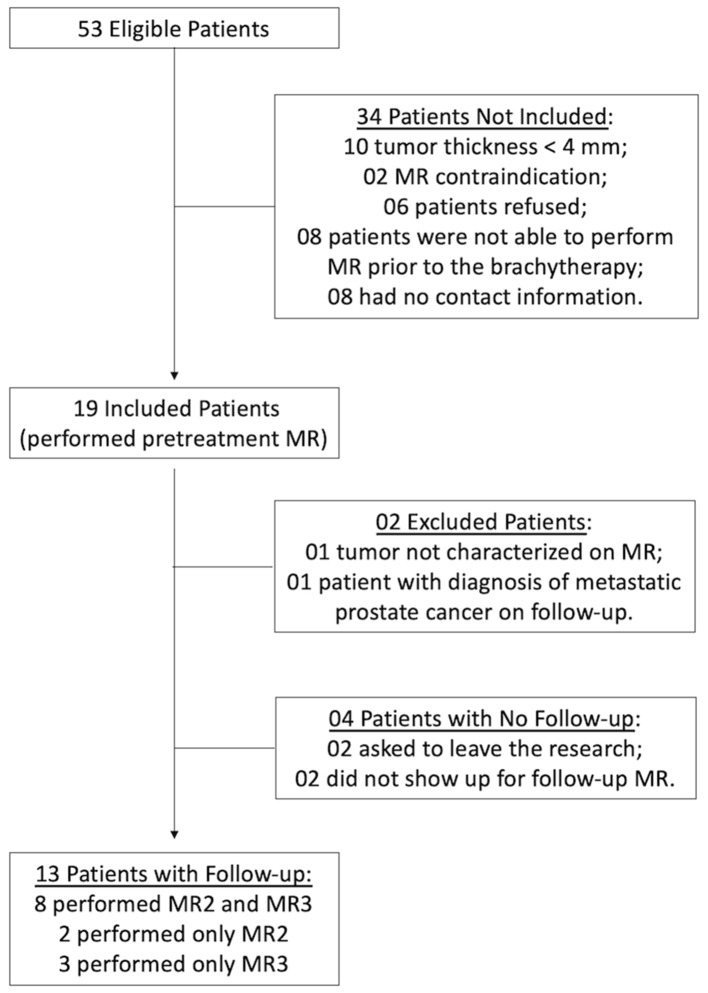
Flowchart of selection and inclusion criteria for patients in the study.

This prospective, single-center study, approved by the institutional research ethics committee, included patients diagnosed with choroidal melanoma and selected for brachytherapy, from July 2018 to June 2019. Exclusion criteria were: patients with tumor thickness less than 4 mm, due to technical difficulty to perform DW-MRI evaluation in these cases; patients with contraindications for MRI (e.g., presence of pacemaker, metallic prosthesis, claustrophobia, etc); and those who had no follow-up at the institution. In the study period, 53 patients underwent brachytherapy for choroidal melanoma at the institution, making them eligible for inclusion in the study. Of these, 34 patients were not included for various reasons (Figure 1). The remaining 19 patients were included in the study and underwent the first MRI exam before brachytherapy. Of these, two patients were excluded from the follow-up: one had a very small tumor that was not well characterized at DWI; the other patient had a final diagnosis of prostate adenocarcinoma metastasis. In addition to these, four patients did not undergo follow-up examinations (Figure 1). Therefore, 13 patients underwent follow-up MRI examinations, of which eight underwent three MRI exams and five patients underwent only two exams.

After agreeing to participate in the study and signing the informed consent form, three MRI exams were proposed for each patient: (MR1) before brachytherapy; (MR2) 1–4 months after treatment; (MR3) 5–8 months after treatment. Because most patients were from other cities or states, the follow-up exams were scheduled according to the patient's consultation dates at the institution.

The brachytherapy protocol at the institution uses episcleral radioactive plaques with iodine-125 (I-125) or ruthenium-106 (Ru-106). The radiation dose was programmed by the physics and radiation oncology departments based on recommendations by the American Brachytherapy Society (7). The choice of the plaque diameter was made by adding 2–4 mm to the largest tumor diameter to have a safety margin of at least 1–2 mm on each side. A dose of 85 Gy was delivered by a minimum dose rate of 0.6 Gy/h at the lesion apex. A margin of 1 millimeter corresponding to sclera thickness was added to the true height value. Dose to the base was then calculated and limited to a range between 250 and 400 Gy, to follow our institutional protocol. Plaques were surgically placed and removed in a special operating room, with radiological protection, under general anesthesia. The surgical procedure consisted of: indirect binocular ophthalmoscopy to locate the lesion, transpupillary transillumination to show the tumor margins, demarcation of these margins in the episcleral tissue with a surgical pen, positioning of the plaque in correct alignment with the tumor, and suture of the plaque in the scleral tissue by progressing points positioned on the plate handles.

Local tumor control was evaluated by ophthalmological monitoring with fundoscopy and ultrasound. For evaluation of the local therapeutic response, local tumor control was defined as stabilization or reduction of the lesion dimensions on ultrasound after treatment. Ocular ultrasonography was performed with high frequency probes (10–50 mHz), measuring the lesion in three axes, including maximum thickness and basal diameters. In the absence of local tumor control after the initial treatment, a second treatment with brachytherapy may have been indicated before surgical treatment.

**Figure 2:**
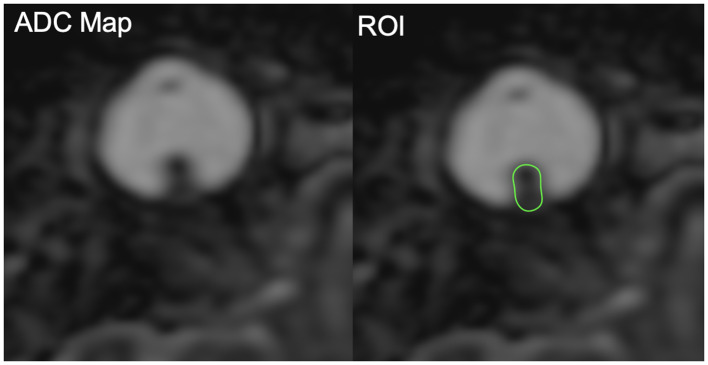
Example of region of interest (ROI) placement covering the whole tumor on ADC maps.

MR images were obtained in a 1.5T device (Achieva, Philips Healthcare, Best, Netherlands), with non-enhanced T1 and T2 weighted multiplanar sequences, using a dedicated coil: Axial T1 (TR/TE: 536/25 ms; Matrix: 200 × 198; Slice thickness: 2 mm; FOV: 120 mm; Acquisition time: 2:16); Axial T2 SPAIR (TR/TE: 4132/90 ms; Matrix: 220 × 213; Slice thickness: 1.82 mm; FOV: 120 mm; Acquisition time: 3:22); Axial Balance (TR/TE: 6.0/3.0 ms; Matrix: 308 × 307; Slice thickness: 0.6 mm; FOV: 180 mm; Acquisition time: 2:10); Coronal T2 (TR/TI: 4144/180 ms; TE: 80 ms; Matrix: 188 × 179; Slice thickness: 3 mm; FOV: 120 mm; Acquisition time: 3:35). The DW-MRI sequence was performed using the ASSET echo-planar imaging (EPI) technique in the axial plane (TR/TI: 5142/165 ms; TE: 76 ms; Matrix: 72 × 55; Slice thickness: 2 mm; FOV: 140 mm; Acquisition time: 6:51). The sensitization of the diffusion gradients was applied in two orthogonal directions with b values of 0 and 1000 s/mm^2^. The DWI sequence was post-processed using Osirix software to obtain apparent diffusion coefficient (ADC) maps. For the patients included in the study, MRI images were evaluated by a single radiologist, with the purpose of adequately characterizing the lesion, in addition to determining their behavior in the diffusion sequence and calculating the ADC values. A region of interest (ROI) was drawn to cover the whole tumor on ADC maps and used to calculate mean, median, minimum, and maximum ADC values (Figure 2).

Statistical analysis was performed using SPSS software version 20.0. Frequencies and percentages were used to describe categorical variables, and mean, standard-deviation (SD) and range were used to describe continuous variables. The size of the main tumor was determined by measurement of the major axes, as assessed by ultrasound and MRI. Differences in ADC values obtained before and after brachytherapy were compared between responders and nonresponders using the Mann-Whitney non-parametric test. Results that had a probability of type I error less than or equal to 5% (*p* ≤ 0.05) were considered statistically significant.

## Results

The mean age of the included patients (*n* = 17) was 57 years (standard deviation [SD]: 15.5 years), ranging from 24 to 78 years, and nine patients (52.9%) were male. The tumor was found in the right eye in 9 patients (52.9%) and in the left eye in 8 patients (47.1%). The most frequent location was the upper temporal quadrant in 10 cases (58.8%), followed by the lower temporal quadrant in 4 cases (23.5%), lower nasal, upper nasal and lower quadrants in 1 case each (5.9 %). On ocular ultrasound, the mean thickness of the tumors was 6.3 mm (SD: 2.0 mm), ranging from 4 to 9 mm, and the mean base diameter was 11.5 mm (SD: 2.3 mm), ranging from 7 to 15 mm. At initial MRI, the mean thickness of the tumors was 5.9 mm (SD: 2.1 mm), ranging from 3 to 10 mm, and the mean diameter of the base was 10.2 mm (SD: 2.2 mm), ranging from 7 to 14 mm. The mean ADC values from the initial MRI exam was 0.96 **×** 10^−3^ mm^2^/s (SD: 0.25 × 10^−3^ mm^2^/s), ranging from 0.49 to 1.55 × 10^−3^ mm^2^/s.

**Figure 3:**
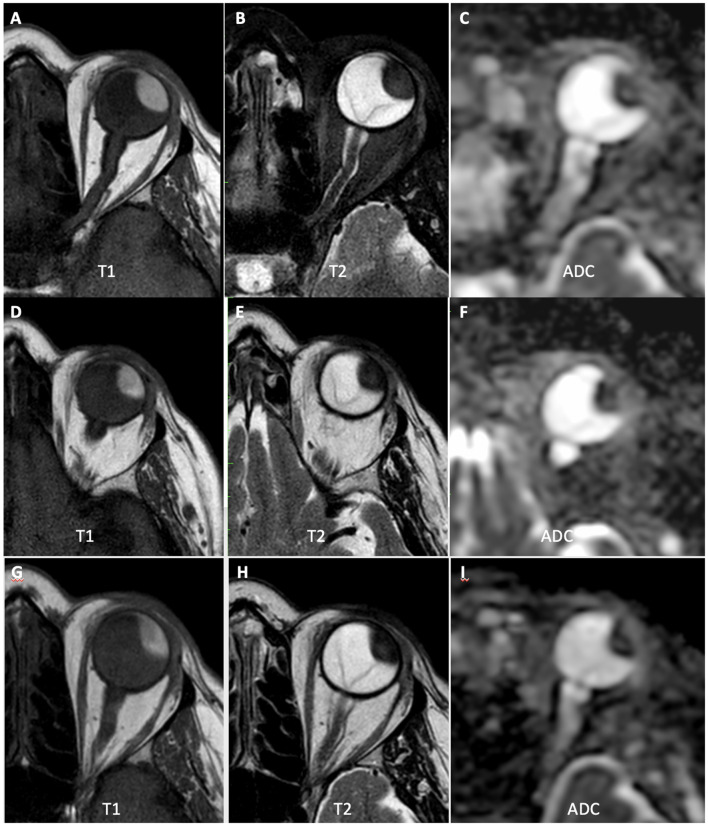
A 66-year-old patient with choroidal melanoma in the left eye (upper temporal quadrant, measuring 15 × 14 × 9 mm), was treated with brachytherapy. **(A–C)** MR1 showing tumor with high signal at T1-weighted images, low signal at T2-weighted images, and restricted diffusion on the ADC map (mean ADC: 0.90 × 10^−3^ mm^2^/s). **(D–F)** MR2 showing that the tumor had similar morphological characteristics and dimensions, but with a slight increase in ADC values compared to the initial examination (mean ADC: 0.97 × 10-3 mm2/s). **(G–I)** MR3 showing a slight reduction in tumor dimensions, with further increase in ADC values (mean ADC: 1.20 × 10^−3^ mm^2^/s).

**Figure 4:**
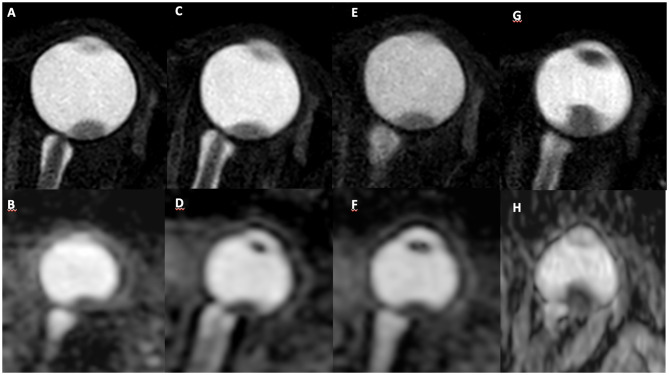
A 59-year-old patient with choroidal melanoma in the left eye (upper temporal quadrant, measuring 9 × 4 mm), was submitted to brachytherapy. Pretreatment MR images: **(A)** axial T2-weighted sequence and **(B)** axial ADC map, with mean ADC of 1.241 × 10^−3^ mm^2^/s. MR images collected 3 months after treatment in the same sequences demonstrate stability of lesion size **(C)** and decreased ADC values **(D)** (mean ADC: 1.007 × 10^−3^mm^2^/s). MR images 6 months after treatment show stability of the lesion size **(E)** and ADC values **(F)**. MR images collected 9 months after treatment show an increase in lesion dimensions **(G)** (13 × 9 mm) and decreased ADC values **(H)** (mean ADC: 0.794 × 10^−3^mm^2^/s).

Considering all patients who underwent follow-up exams (*n* = 13), no significant differences were observed in the means of tumor size or ADC values between MR1, MR2, and MR3. Most patients (*n* = 8; 61.5%) showed stability (variation less than 10%) or increased mean ADC values after treatment (Figure 3). The mean time of clinical follow-up after brachytherapy ranged from 4 to 15 months, with 11 patients (84.6%) having been followed for more than 6 months. Most patients (*n* = 11; 84.6%) showed stability of the lesion dimensions on ocular ultrasound after brachytherapy. However, two patients showed signs of disease progression during follow-up:

- One patient presented an increase in tumor size 7 months after brachytherapy (from 11 × 6 mm to 13 × 7 mm), so a second brachytherapy was performed. This patient did not undergo MR2. There was a slight increase in the dimensions of the lesion (9 × 5 mm to 10 × 6 mm) and a reduction in the mean ADC values between MR1 (0.91 × 10^−3^mm^2^/s) and MR3 (0.69 × 10^−3^mm^2^/s).
- One patient presented an increase in tumor size 9 months after brachytherapy (from 9 × 4 mm to 13 × 9 mm) and was submitted to enucleation. There was no significant change in the lesion size between MR1, MR2, and MR3; however, there was a reduction in mean ADC values between MR1 (1.24 × 10^−3^mm^2^/s) and MR2 (1.01 × 10^−3^mm^2^/s) (Figure 4).

Considering the variations in ADC values between the pre-treatment MRI and the first post-treatment MRI, there was a significant reduction in the mean ADC in patients who presented disease progression (*p* = 0.02); there was no significant difference in the median, minimum, or maximum ADC values (Table 1).

**Table 1:** Variation in ADC values (×10^−3^mm^2^/s) between pre and post-treatment MRI given in mean (range).

	**Total (*n* = 13)**	**Stable (*n* = 11)**	**Progression (*n* = 2)**	***p***
Mean ADC	−0.02 (−23.90; 26.42)	3.86 (−15.89; 26.42)	−21.38 (−23.90; −18.86)	**0.02**
Median ADC	−0.85 (−36.01; 52.28)	1.45 (−36.01; 52.28)	−13.49 (−15.28; −11.69)	0.51
Minimum ADC	4.77 (−100; 226.32)	6.08 (−100; 226.32)	−0.47 (−35.11; 34.16)	1.00
Maximum ADC	−8.60 (−46.62; 11.98)	−4.81 (−24.80; 11.98)	−29.44 (−46.62; −12.26)	0.15

## Discussion

There is disagreement in the literature regarding the best criteria to assess prognosis in patients with choroidal melanoma. Damato et al. (10) and Deparis et al. (28) created an online tool to estimate the prognosis of uveal melanoma patients, including clinical, pathological, and genetic risk factors, which has already been validated in other populations (9, 26). Currently, the main risk factors considered for the development of metastasis are the size of the tumor at diagnosis and the gene expression profile (29). However, the way to obtain material for performing genetic tests is through intraocular biopsy. The most common procedure for this is fine needle aspiration biopsy (FNAB), either transscleral or transvitreous depending on the location of the tumor (30–32). The success rate of this procedure is very high (33). Complications are uncommon and include vitreous hemorrhage, retinal detachment and, rarely, extraocular extension (34). There have been reports of tumors remaining implanted in the vitreous after FNAB (35).

There is also disagreement in the literature concerning the role of local treatment in choroidal melanoma prognosis, and how to assess the response to treatment. There have been patients submitted to enucleation who end up developing metastasis, and there are also those who are left untreated who develop no complications. Even in patients with small choroidal melanoma treated with brachytherapy, the risk of distant metastasis is about 9% (36).

In the present study, we evaluated the use of DW-MRI in the response evaluation of patients with choroidal melanoma undergoing brachytherapy. Other authors have demonstrated the use of other MRI sequences to characterize melanomas, such as the T2 FLAIR sequence and post-contrast sequences to assess vascularization and perfusion of the lesion (37, 38). In our work, we used a more objective protocol and intravenous MRI contrast was not administered for two reasons. First, because the main objective of the work was to evaluate the DW-MRI sequence, which does not require contrast administration. Second, to facilitate the exam logistics, reduce the cost and time of acquisition, and avoid complications or adverse effects related to venipuncture or contrast administration.

One of the limitations of DW-MRI to evaluate intraocular tumors is the poor spatial resolution, which makes it difficult to analyze ADC values in very small tumors. For this reason, tumors <4 mm in thickness were not included in this study. The incorporation of MRI devices with a larger magnetic field and the development of specific coils can improve the resolution of this method, allowing better characterization of intraocular lesions and evaluation of even smaller lesions (39–41).

The mean ADC values before treatment observed here (0.997 ± 0.248 × 10^−3^ mm^2^/s) were similar to the values described in other studies in the literature that used similar b-values for ADC calculation (ranging from 0.891 ± 0.172 to 1.180 ± 0.160 × 10^−3^ mm^2^/s) (22, 24–27, 42). In our study, there was a large variation among ADC values before treatment (ranging from 0.49 to 1.55 × 10^−3^mm^2^/s), which can reflect differences in cellularity between different melanomas. Most prior studies did not report the ADC values range of choroidal melanomas; in the only study that reported it, the range was between 0.75 and 1.52 × 10^−3^mm^2^/s (42).

Numerous studies in different tumor types have shown that ADC values changes after cancer treatments, including radiation therapy, providing early response assessment (43–45). While an increase in ADC values during therapy is frequently related to reduced cellularity in the tumor caused by treatment-induced cell death, a decrease in ADC values suggests poor response to treatment. In the present study, we observed a statistically significant reduction in the mean ADC values between pre-treatment MRI and the first post-treatment MRI in patients who presented disease progression, when compared to patients who did not show progression. In a study that evaluated DW-MRI after treatment with proton beam radiotherapy, Foti et al. also demonstrated that an early increase in ADC values has a significant correlation with the tumor regression rate (25, 27).

The results of this work must be considered in the context of some limitations. First, only a small number of cases were evaluated due to patient refusals and lack of follow-up. Our relatively short follow-up time and few cases with progression were compatible with the literature, but made it impossible to carry out more detailed statistical analyses. We used one single radiologist evaluation to standardize the ROI measurements between pre and post treatment exams, thus it was not possible to assess interobserver agreement in this study. Despite these limitations, this study demonstrates that DW-MRI has the potential to be used as an early indicator of therapeutic response in patients with choroidal melanoma treated with brachytherapy. The development of non-invasive methods that allow early assessment of response in these cases can have a direct impact on current clinical practice.

In conclusion, DW-MRI is a promising method for monitoring patients with choroidal melanoma; reduction in the mean ADC values between pre-treatment MRI and the first post-treatment MRI may be related to the lack of response to brachytherapy and increased risk of disease progression. Further studies with larger samples are recommended to confirm these findings.

## Data Availability Statement

The datasets generated for this study are available on request to the corresponding author.

## Ethics Statement

The studies involving human participants were reviewed and approved by Fundação Antônio Prudente-A.C.Camargo Cancer Center. The patients/participants provided their written informed consent to participate in this study.

## Author Contributions

FB, AB, MC, and RC gave substantial contributions to the conception or design of the work. JS performed data acquisition. FB and AB analyzed the data and drafted the manuscript. All authors revised it critically and provides approval for publication of the content.

## Conflict of Interest

The authors declare that the research was conducted in the absence of any commercial or financial relationships that could be construed as a potential conflict of interest.
